# Longitudinal Patient-Reported Symptom Change Patterns and Prediction of Future Health-Related Quality of Life in Childhood Cancer Survivors: A Machine Learning Approach from the Childhood Cancer Survivor Study and the St. Jude Lifetime Cohort

**DOI:** 10.3390/cancers18101546

**Published:** 2026-05-10

**Authors:** Farideh Bagherzadeh-Khiabani, Kevin R. Krull, Shizue Izumi, Sedigheh Mirzaei, Tiange Zheng, Jose Miguel Martinez Martinez, Kirsten K. Ness, Gregory T. Armstrong, Melissa M. Hudson, Leslie L. Robison, Yutaka Yasui, I-Chan Huang

**Affiliations:** 1 School of Public Health, University of Alberta, Edmonton, AB T6G 2R3, Canada; bagherza@ualberta.ca (F.B.-K.); jmiguelmartinezmartinez@gmail.com (J.M.M.M.); 2 Department of Psychology and Biobehavioral Sciences, St. Jude Children’s Research Hospital, Memphis, TN 38105, USA; kevin.krull@stjude.org; 3 Faculty of Data Science, Shiga University, Hikone 522-8522, Japan; 4 Department of Epidemiology and Cancer Control, St. Jude Children’s Research Hospital, Memphis, TN 38105, USA; 5 Department of Biostatistics, St. Jude Children’s Research Hospital, Memphis, TN 38105, USA; tiange.zheng@stjude.org; 6 Public Health Research Group, University of Alicante, 03690 San Vicente del Raspeig, Alicante, Spain; 7 Department of Oncology, St. Jude Children’s Research Hospital, Memphis, TN 38105, USA

**Keywords:** childhood cancer survivors, health-related quality of life, longitudinal patterns, patient-reported symptoms, prediction

## Abstract

Background: Adult survivors of childhood cancer face a significant risk for treatment-related late effects that may impair health-related quality of life (HRQoL). Incorporating longitudinal changes in patient-reported symptoms beyond treatment-based risk factors may enhance the prediction of HRQoL. Methods: Survivors (*n* = 576) dually enrolled in the St. Jude Lifetime Cohort Study and Childhood Cancer Survivor Study reported 37 symptoms across 10 domains at three time points over 20 years to ascertain longitudinal symptom change patterns. HRQoL was subsequently assessed using SF-36 scores. Prediction models were developed using Bayesian Information Criterion Elastic Net (BIEN), first including demographic, diagnosis, and treatment variables, then adding symptom change patterns. Prediction of suboptimal HRQoL (score < 40) was evaluated using 10-fold cross-validated area under the receiver operating characteristic curve values (AUC). Results: Participants (median baseline age 26.7 years, 52% female, 90% non-Hispanic white, 41% leukemia, and 30% Hodgkin/non-Hodgkin lymphoma survivors) most frequently reported symptom domains of sensory, pain, and anxiety (50–60% at any time point), followed by depression and memory (40–50%). Consistent absence throughout follow-up was the most common pattern (41.7–98.1%), while patterns requiring symptom presence at ≥1 time point were less common (0.0–16.7%), and persistent presence throughout follow-up was rare (0.0–6.8%). Across 10 SF36-HRQoL scores, symptom-enhanced models improved prediction over non-symptom models (AUCs 0.75–0.85 vs. 0.56–0.66; *p*-values < 0.001). Conclusions: Longitudinal symptom change patterns substantially improved future HRQoL prediction, achieving prediction accuracy that may be of clinical effectiveness. This supports regular symptom assessment and further research towards symptom-informed risk stratification in survivorship care.

## Simple Summary

Survivors of childhood cancer are at high risk of experiencing health problems later in life that affect their long-term physical and emotional well-being. These experiences are not fully predictable by traditional medical measures such as treatments used for childhood cancer. In this study, by looking at how symptoms appear, disappear, or persist over time, along with the traditional medical measures, we aimed to better understand and predict who may be at high risk of poor health later in life. By achieving this aim, researchers and healthcare providers may be able to recognize vulnerable survivors earlier, which in turn could assist in timely support and interventions, helping survivors enjoy a healthier adult life.

## 1. Introduction

Advances in childhood cancer treatment have increased five-year survival rates substantially, from around 20% in 1950–1954 to over 85% currently in the US [1,2,3]. With this success comes growing concern about the long-term adverse effects of cancer and cancer therapy [4,5,6,7]. In addition to treatment-related risks, early identification of modifiable predictors associated with late effects may provide opportunities for timely, targeted interventions that support healthier aging among survivors [8,9,10].

In adult cancer survivorship research, routine collection of symptoms has been associated with meaningful benefits, including better HRQoL, fewer emergency room visits, and improved survival compared with standard care [11,12,13]. These gains may arise from earlier recognition of emerging problems in the care continuum, which can prompt more effective discussions between patients and healthcare providers, enhance identification of underlying health conditions and their determinants, support patient-centered decision-making, and facilitate timely and appropriate interventions such as symptom management counseling, supportive medications, diagnostic evaluation, treatment adjustments, and specialty referrals [11,12,13]. Despite growing evidence of their value, symptom assessment is not routinely incorporated into clinical trials, survivorship or primary care visits, and research analyses [14,15,16].

Prior cancer research utilizing symptom data as predictors has largely focused on individual symptoms (e.g., pain, fatigue, or psychological distress) or aggregate symptom burden assessed at a single time point. Such approaches potentially overlook dynamic changes as survivors age, during which both health status and perception of well-being evolve over time [15,16,17,18]. The prognostic value of longitudinal symptom changes remains insufficiently explored in childhood cancer survivors.

Addressing this gap requires flexible modeling strategies. Machine learning methods have shown considerable promise for predicting health outcomes when numerous predictors are considered [19], enabling data-driven identification of the most informative subset of predictors and allowing predictor–outcome relationships to be learned from the data rather than specified a priori.

This study aims to determine whether longitudinal changes in symptoms improve the prediction of future HRQoL in adult survivors of childhood cancer. Utilizing longitudinal data over 20 years from survivors co-enrolled in the Childhood Cancer Survivor Study (CCSS) [20] and St. Jude Lifetime Cohort Study (SJLIFE) [21], we employed machine learning variable selection and coefficient estimation techniques to predict future HRQoL scores from candidate longitudinal patterns of 37 patient-reported symptoms measured at three time points and to identify a parsimonious, informative subset of symptom change patterns.

## 2. Materials and Methods

### 2.1. Study Population

This study included 576 five-year survivors of childhood cancer who participated in both CCSS and SJLIFE, through which symptom data were collected at three time points (T1 [study baseline], T2, T3) and the HRQoL outcome was collected after T3 symptom assessment. Eligibility criteria required participants to be at least 18 years of age at T1 symptom assessment, to have been diagnosed with cancer before age 21, to have completed patient-reported (rather than proxy/caregiver-reported) symptom questionnaires at three time points through CCSS/SJLIFE, and to have subsequently completed a self-reported HRQoL questionnaire through SJLIFE.

CCSS, initiated in 1994, is a retrospectively constructed cohort with prospective follow-up of 25,735 five-year survivors diagnosed between 1970 and 1999 and treated for childhood cancer at 31 institutions across North America. SJLIFE, initiated in 2007, is a retrospectively constructed cohort with prospective follow-up of more than 6600 five-year survivors diagnosed after 1962 and treated at St. Jude Children’s Research Hospital. Both cohorts assess occurrences of late effects of childhood cancer and its treatment, with ongoing prospective follow-up throughout adulthood. Details of the studies have been published previously [20,21].

### 2.2. Measurement

#### 2.2.1. HRQoL Outcomes

HRQoL was assessed using the 36-Item Short Form Survey (SF-36) [22], a validated questionnaire that captures eight domains: physical functioning, role limitations due to physical health problems, bodily pain, general health perceptions, vitality, social functioning, role limitations due to emotional health problems, and mental health. Additionally, the physical component summary (PCS) and the mental component summary (MCS) are calculated using an established weighting schema [23]. All ten scores are standardized to a mean of 50 and a standard deviation of 10 based on general population norms, with higher values indicating better HRQoL. For performance reporting, HRQoL scores were dichotomized using a cut-off of 40 (1 standard deviation below the general population mean) to define suboptimal HRQoL, a threshold commonly used to identify clinically meaningful impairment [24,25,26]. The median calendar year for completing the HRQoL survey was 2016 (IQR: 2015–2017) (Supplementary Figure S1).

#### 2.2.2. Non-Symptom Predictors

Non-symptom predictors included demographic characteristics, cancer diagnosis, and cancer treatment exposures. Demographic predictors comprised age at the T1 symptom survey, time between T1 symptom and HRQoL surveys, age at T3 symptom survey, time between T3 symptom and HRQoL surveys, age at cancer diagnosis, sex, race/ethnicity (non-Hispanic white vs. others), and educational attainment at T1 (college graduate or higher vs. less than college).

Cancer diagnoses (leukemia, Hodgkin lymphoma, non-Hodgkin lymphoma, osteosarcoma, Wilms tumor, neuroblastoma, central nervous system tumors) were abstracted from medical records by trained research staff. Treatment exposures within the first five years after primary cancer diagnosis were also abstracted from the medical records, including chemotherapy (yes/no) for specific agents (methotrexate, intrathecal methotrexate, high-dose methotrexate, cytarabine, intrathecal cytarabine, high-dose cytarabine, bleomycin, alkylating agent, anthracycline, corticosteroid, plant alkaloid, platinum), radiation therapy (yes/no) to specific sites (brain, neck, chest, abdomen, pelvis), surgical procedures, including amputation (yes/no), and other surgery (yes/no).

#### 2.2.3. Symptom Predictors

Symptom predictors were derived from responses to 37 items assessing the presence/absence of an individual symptom (symptom items). These items, originally developed in CCSS and subsequently used in CCSS/SJLIFE [27], include 19 designed by CCSS and 18 adapted from the Brief Symptom Inventory-18. Prior work has established robust structural validity of the underlying measurement model using standard fit indices and demonstrated that these symptom items capture meaningful variation associated with prior cancer treatments [27]. Survivors completed three symptom surveys over two decades. The median (IQR) calendar years of completion were 1996 (1996–2008), 2008 (2007–2010), and 2013 (2013–2014), respectively, corresponding to the administration period of the three surveys (Supplementary Figure S1). Each survey required multiple years to complete due to the large number of participants, with repeated contacts conducted through multiple modes. Most survivors responded close to the intended survey administration timing, with some variation, and thus, the intervals between surveys are approximately consistent for the majority of survivors.

### 2.3. Statistical Analysis

The analysis framework consisted of five steps. First, we preprocessed the data (Section 2.3.1). Second, we performed feature engineering to encode longitudinal symptom behavior across three assessments into predefined symptom change pattern indicators (Section 2.3.2). This pattern-definition step was a manual, deterministic procedure, rather than a data-driven one, and was designed to convert repeated symptom measures into structured candidate predictors for downstream modeling. Third, these symptom patterns, along with non-symptom predictors, were entered into a machine learning procedure to identify an informative combination of predictors and estimated coefficients (Section 2.3.3). Fourth, we conducted post-selection inference and assessed the stability of selected predictors (Section 2.3.4). Finally, we evaluated prediction performance using cross-validation (Section 2.3.5). Details of these steps are described below, and a schematic overview of the analytic workflow is shown in Supplementary Figure S2.

#### 2.3.1. Preprocessing

Missing symptom responses at each time point, which accounted for less than 2% of all symptom predictors, were replaced with the value from the preceding time point, or if unavailable, from its successive time point. For survivors who did not provide a given symptom at any of the three time points, the symptom was considered absent, consistent with the predominant observation in the cohort.

#### 2.3.2. Feature Engineering: Longitudinal Symptom Change Patterns

**Figure 1:**
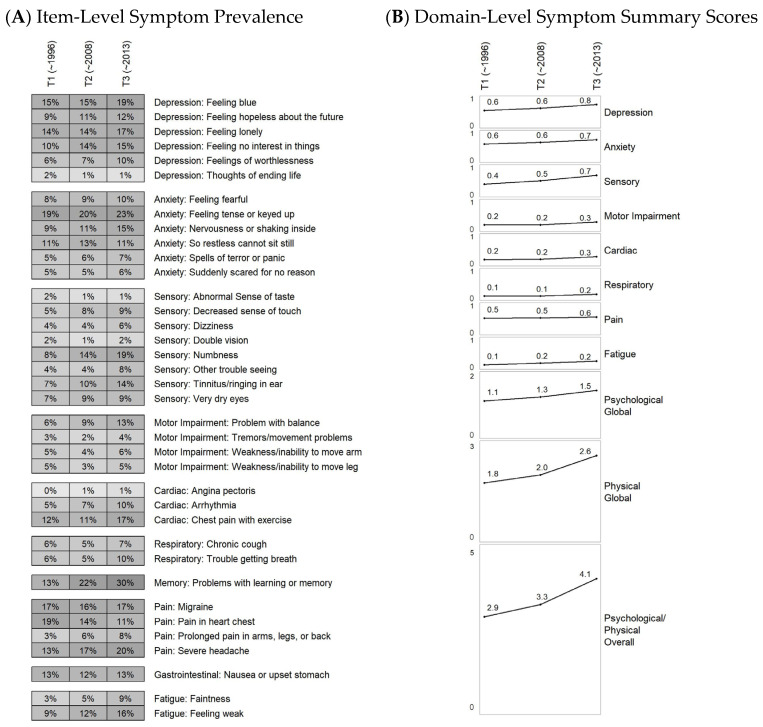
Item-level symptom prevalence and domain-level symptom summary scores across three time points. (Panel (**A**)) displays symptom item prevalence (%) across three assessment time points, grouped by symptom domain, with darker shading indicating higher prevalence. (Panel (**B**)) summarizes trends in the average number of symptoms within 8 individual and 2 global domains, and an overall summary experienced by survivors. The psychological global domain comprises symptom items corresponding to the anxiety and depression domains, and the physical global domain comprises symptom items from the remaining eight domains. Domain-level and overall summaries reflect counts of contributing symptom items and therefore vary in scale according to the number of items included. Together, these panels illustrate both item-level and aggregated domain-level trends over time.

To construct longitudinal symptom patterns for modeling, we first generated cross-sectional symptom summaries from the 37 individual symptom items (see (A) below), then derived longitudinal symptom change patterns utilizing both the original symptom items and the derived cross-sectional symptom summaries (see (B) below). These steps are illustrated in Supplementary Figure S3:

(A) Cross-sectional symptom summaries: To generate cross-sectional symptom summaries, the 37 symptom items were categorized into individual and global domains using a previously established classification [27]. Symptom items were first arranged into 10 individual domains: depression (thoughts of ending life, feeling lonely, feeling blue, feeling no interest in things, feeling hopeless about the future, and feelings of worthlessness), anxiety (nervousness or shaking inside, suddenly scared for no reason, feeling fearful, feeling tense or keyed up, spells of terror or panic, and so restless cannot sit still), sensory (decreased sense of touch, tinnitus/ringing in ear, dizziness, double vision, other trouble seeing, very dry eyes, abnormal sense of taste, and numbness), motor (problem with balance, tremors/movement problems, weakness/inability to move arm, and weakness/inability to move leg), cardiac (arrhythmia, angina pectoris, and chest pain with exercise), respiratory (chronic cough and trouble getting breath), memory (one symptom item of problems with learning or memory), pain (migraine, pain in heart chest, severe headache, and prolonged pain in arms, legs, or back), gastrointestinal (nausea or upset stomach), and fatigue (faintness and feeling weak). Individual symptom domains were further grouped into two global domains: “psychological domain” (symptom items corresponding to anxiety and depression domains) and “physical domain” (symptom items corresponding to the eight remaining domains). After organizing the symptoms into individual and global domains, cross-sectional symptom summaries were created by counting the number of symptoms present overall and within each individual and global domain. The memory and gastrointestinal domains were excluded from summarization because each contained only one symptom item. This process yielded 11 additional cross-sectional symptom measures, including the overall summary of all 37 symptom items, the global psychological domain of the 12 psychological symptoms, the global physical domain of the 25 physical symptoms, and eight for the eight individual domains containing more than one symptom item. These 11 aggregated symptom measures brought the total number of cross-sectional symptom measures to 48 at each of the three time points (Figure 1 and Supplementary Table S1).
(B) Longitudinal symptom change patterns were engineered from the 48 cross-sectional symptom measures to capture clinically meaningful trajectories that may be associated with future suboptimal HRQoL. This feature engineering approach leverages domain expertise to construct meaningful predictors from observed data. Using this approach, we defined 10 meaningful longitudinal symptom change patterns (P1–P10). These patterns describe changes in symptom presence across the three time points and are not mutually exclusive:
  - P1. Early Escalation: absent at T1, present at T2
    (i.e., a subject did not report the symptom at T1 but did at T2).
  - P2. Late Escalation: absent at T2, present at T3
    (i.e., a subject did not report the symptom at T2 but did at T3).
  - P3. Early Resolution: present at T1, absent at T2
    (i.e., a subject reported the symptom at T1 but not at T2).
  - P4. Late Resolution: present at T2, absent at T3
    (i.e., a subject reported the symptom at T2 but not at T3).
  - P5. Persistent Presence: present at T1, T2, and T3
    (i.e., a subject reported the symptom at T1, T2, and T3).
  - P6. Early Limited Persistence: present at T1 and T2, absent at T3
    (i.e., a subject reported the symptom at T1 and T2 but not at T3).
  - P7. Late Limited Persistence: absent at T1, present at T2 and T3
    (i.e., a subject did not report the symptom at T1 but did at T2 and T3).
  - P8. Consistent Absence: absent at T1, T2, and T3
    (i.e., a subject did not report the symptom at T1, T2, or T3).
  - P9. Early Limited Absence: absent at T1 and T2, present at T3
    (i.e., a subject did not report the symptom at T1 and T2 but did at T3).
  - P10. Late Limited Absence: absent at T2 and T3, present at T1
    (i.e., a subject did not report the symptom at T2 and T3 but did at T1).

Patterns P1–P10 were applied to each of the 37 individual symptoms (Supplementary Figure S3), and analogous patterns were defined for the 11 cross-sectional symptom summaries. For escalation and resolution patterns, cross-sectional summaries were treated as continuous variables, defined by the number of symptoms reported (ranging from 0 to the maximum possible within each summary), thereby yielding continuous measures of change over time. This approach allows varying degrees of symptom burden across individual and global domains to inform the models. In contrast, for the persistence and absence patterns, cross-sectional summaries were coded as binary variables, indicating the presence or absence of any symptom within a summary (i.e., symptom count > 0) at the relevant time points.

The generated symptom change patterns represent various possible combinations of symptom presence or absence across the three time points (T1–T3), designed to capture longitudinal trajectories of symptom status over follow-up (e.g., escalation, resolution, and persistence), and thus, to inform subsequent HRQoL outcomes. Specifically, patterns reflecting persistence or escalation of symptoms were hypothesized to be associated with poorer HRQoL, whereas patterns reflecting symptom resolution or consistent absence were hypothesized to be associated with better HRQoL.

#### 2.3.3. Modeling of HRQoL Outcomes Utilizing Non-Symptom Measures and Symptom Change Patterns

For each of the 10 HRQoL scores, prediction modeling was conducted in two stages. The non-symptom model included only demographic characteristics, cancer diagnosis, and treatment exposures, excluding all symptom-related predictors. The symptom-enhanced model incorporated the longitudinal symptom change patterns in addition to the non-symptom predictors.

To identify the optimal combinations of predictors for each HRQoL score and to quantify their associations, we utilized BIEN [28]. BIEN is a data-driven machine learning technique that enables predictor/model selection and parameter inference simultaneously, eliminating the need for a priori hypotheses regarding the set of predictors associated with the outcome. This approach is particularly suited to the present study, given the large number of potential predictors, 480 longitudinal symptom change patterns derived by defining the 10 patterns in each of 48 cross-sectional symptom measures, and the difficulty of formulating clear hypotheses about their relationships with HRQoL. By accounting for the joint effects of a wide range of potential predictors, BIEN enables identifying predictors that may be informative only in combination with others. Detailed information on BIEN can be found in our companion work [28], and the source code is available online [link will be provided upon acceptance]. BIEN consists of five key components, which are briefly described below, and a schematic overview of how these components interact is provided in Supplementary Figure S4.

(1) Predictor set generation (Elastic Net): Using Elastic Net, a penalized regression approach, this component generates a candidate set of predictors for a given pair of hyperparameter values, which jointly determine the magnitude and the form of regularization applied to control model complexity.
(2) Model estimation (maximum likelihood): Utilizing maximum likelihood estimation, this component estimates the strengths of associations corresponding to each candidate predictor set.
(3) Model scoring (Bayesian Information Criterion): Candidate models are evaluated using the Bayesian Information Criterion, which balances goodness of fit and complexity to produce a ranking score.
(4) Model search (truncated grid search): A truncated grid search is conducted to efficiently identify a targeted set of penalty levels and penalty forms to be explored during predictor set generation.
(5) Model refinement (pruning): The selected model is subsequently refined using backward elimination, iteratively removing predictors that do not contribute significantly to the model.

#### 2.3.4. Post-Selection Inference and Stability Assessment

We assessed uncertainty in coefficient estimates and the stability of selected predictors using nonparametric bootstrap resampling with 1000 replicates. To quantify uncertainty in coefficient estimates, we constructed empirical 95% confidence intervals from the bootstrap distribution, assigning a coefficient value of zero in replicates where BIEN did not select the corresponding predictor to reflect non-selection. To evaluate the stability of variable selection, we calculated the bootstrap selection probability for each predictor in the final model as the proportion of bootstrap replicates in which that predictor was selected.

#### 2.3.5. Prediction Performance Evaluation

For each HRQoL outcome, predictive performance was evaluated using the area under the receiver operating characteristic curve (AUC) for the suboptimal HRQoL defined as a score < 40 (i.e., one standard deviation below the general population mean). AUCs were calculated using 10-fold cross-validation, repeating the entire model-building process above 10 times, each time with a different set of 9 out of 10 mutually exclusive, randomly created, equally sized blocks of data, retaining one block for independent prediction evaluation, to provide unbiased estimates of the performance metric AUC.

### 2.4. Analytic Software

All analyses were performed using R version 4.2.1 (R Project for Statistical Computing) [29]. The R function BIEN was used for model fitting [28].

## 3. Results

There were 576 survivors who met the eligibility criteria and were included in this study. They were diagnosed with cancer at a median age of 9.3 years (IQR 4.5–14.1) and were 26.7 years (IQR 23.0–30.4) at T1 symptom assessment and 39.5 years (IQR 34.8–44.6) at T3 symptom assessment. Overall, 52% were women, 90% were non-Hispanic white, and 34% had a college degree or higher (Table 1). Leukemia was the most common diagnosis (41%), followed by Hodgkin lymphoma (20%). Regarding treatment exposure, 89% received chemotherapy, 72% received radiotherapy, and 61% underwent surgery, including 31 survivors (5%) who underwent amputation (Table 1).

Across the three symptom assessments, 91% of survivors reported at least one symptom. Sensory, pain, and anxiety symptom domains were most prevalent, each reported by 50–60% of survivors, followed by depression and memory problems (40–50%). Cardiac, fatigue, gastrointestinal, motor, and respiratory domains were relatively less common (20–40%). Prevalence of each symptom domain was defined as reporting at least one symptom within that domain at any of the three assessments (Figure 1 and Supplementary Table S1).

**Figure 2:**
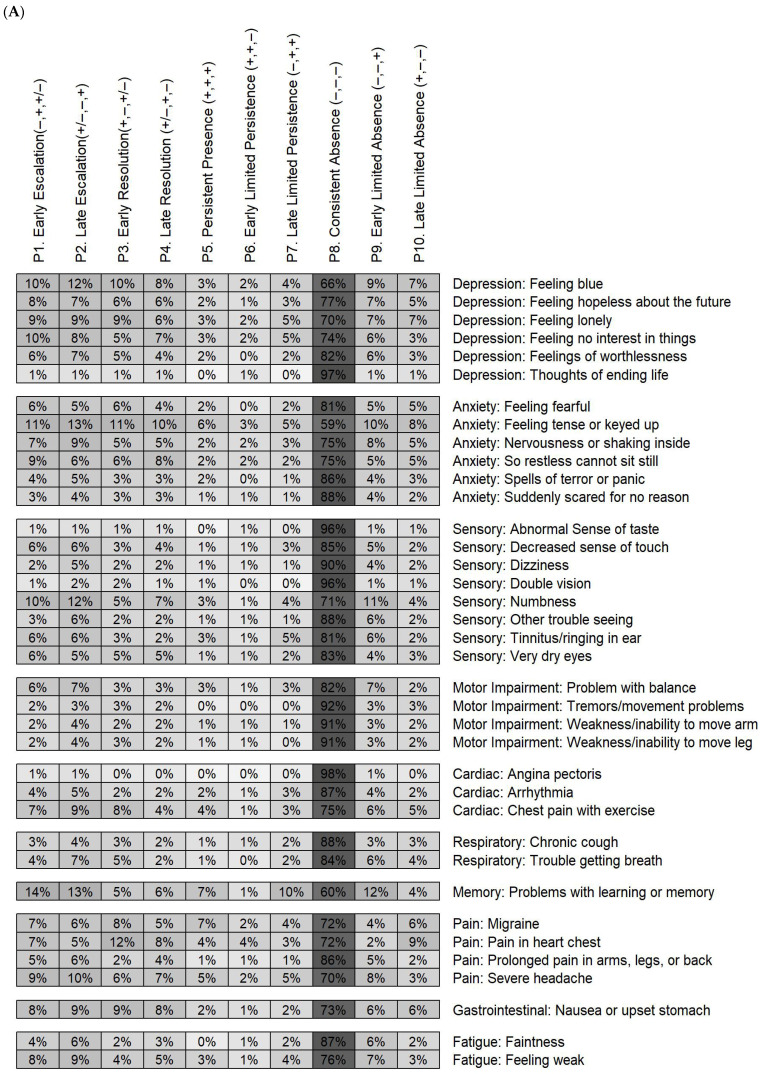

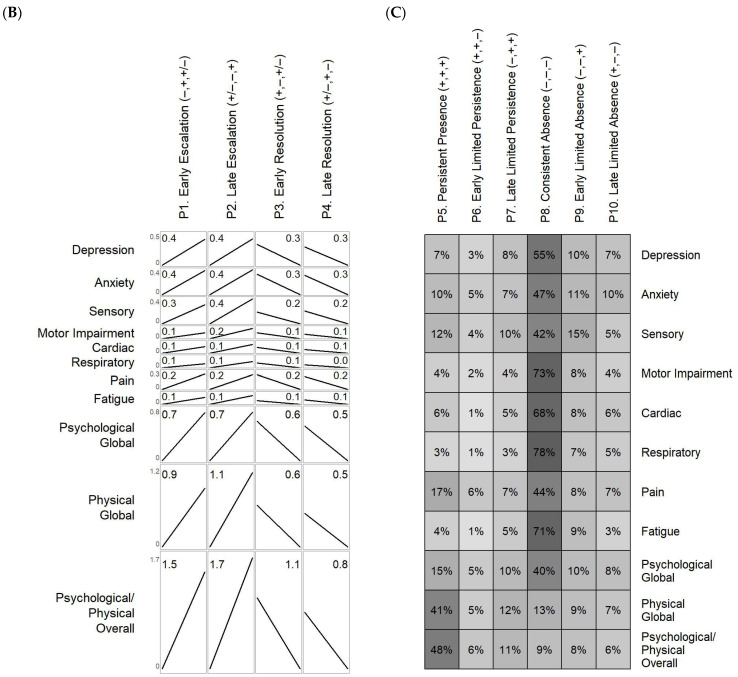
Item-level and domain-level symptom change patterns across three time points. (**A**) Item-level change patterns, (**B**) escalation/resolution patterns for domain-level symptom summary scores, (**C**) persistence/absence patterns for domain-level symptom summary scores. (Panel (**A**)) presents the prevalence (%) of each pattern (P1–P10) across the 37 symptom items, with symptoms grouped by domain; darker shading indicates higher prevalence. (Panel (**B**)) corresponds to P1–P4 and shows the average magnitude of aggregated domain-level change patterns. Values are expressed as slopes to reflect change over time, with positive slopes indicating escalation patterns and negative slopes indicating resolution patterns. Changes are shown relative to zero. (Panel (**C**)) corresponds to P5–P10 and represents the prevalence (%) of domain-level change patterns; darker shading indicates higher prevalence. Together, the three panels provide a complementary view of item-level and aggregated domain-level change patterns. P1–P10 are described as follows: P1. Early escalation (i.e., a subject did not report the symptom at T1 but did at T2). P2. Late escalation (i.e., a subject did not report the symptom at T2 but did at T3). P3. Early resolution (i.e., a subject reported the symptom at T1 but not at T2). P4. Late resolution (i.e., a subject reported the symptom at T2 but not at T3). P5. Persistent presence (i.e., a subject reported the symptom at T1, T2, and T3). P6. Early limited persistence (i.e., a subject reported the symptom at T1 and T2 but not at T3). P7. Late limited persistence (i.e., a subject did not report the symptom at T1 but did at T2 and T3). P8. Consistent absence (i.e., a subject did not report the symptom at T1, T2, and T3). P9. Early limited absence (i.e., a subject did not report the symptom at T1 and T2 but did at T3). P10. Late limited absence (i.e., a subject did not report the symptom at T2 and T3 but did at T1). The symbols “+” and “−” appearing above each panel indicate the presence and absence of a symptom, respectively, at each time point for the corresponding pattern. Darker shading in panels (**A**,**C**) represent a higher observed proportion of the corresponding pattern.

Figure 2 and Supplementary Table S2 summarize survivors’ 10 longitudinal symptom change patterns (P1–P10) across the three time points. Consistent absence (P8) was the most frequent trajectory, with item-specific prevalence ranging from 41.7% to 98.1%. Trajectories involving symptom presence at one or two time points were far less common (symptom-specific prevalence range from 0.0% to 16.7%), with escalation or limited persistence more likely in later adulthood (P2 > P1; P7 > P6), and resolution or limited absence more common in earlier adulthood (P3 > P4; P9 > P10). Persistent presence across all three time points was relatively rare (item-specific prevalence 0.0% to 6.8%).

At the survivor level, defined as exhibiting a given pattern in at least one symptom item, 82% of survivors experienced escalation in ≥1 symptom item, including 58% with early escalation and 66% with late escalation. Similarly, 78% experienced resolution in ≥1 symptom item, including 59% with early resolution and 53% with late resolution. Persistence of ≥1 symptom item across time points was observed in 57% of survivors, including 24% with early limited persistence, 39% with late limited persistence, and 32% with persistent presence across all three time points. Absence in ≥1 symptom item was observed in all survivors, including 62% with early limited absence, 54% with late limited absence, and 100% with consistent absence across all three time points.

**Figure 3:**
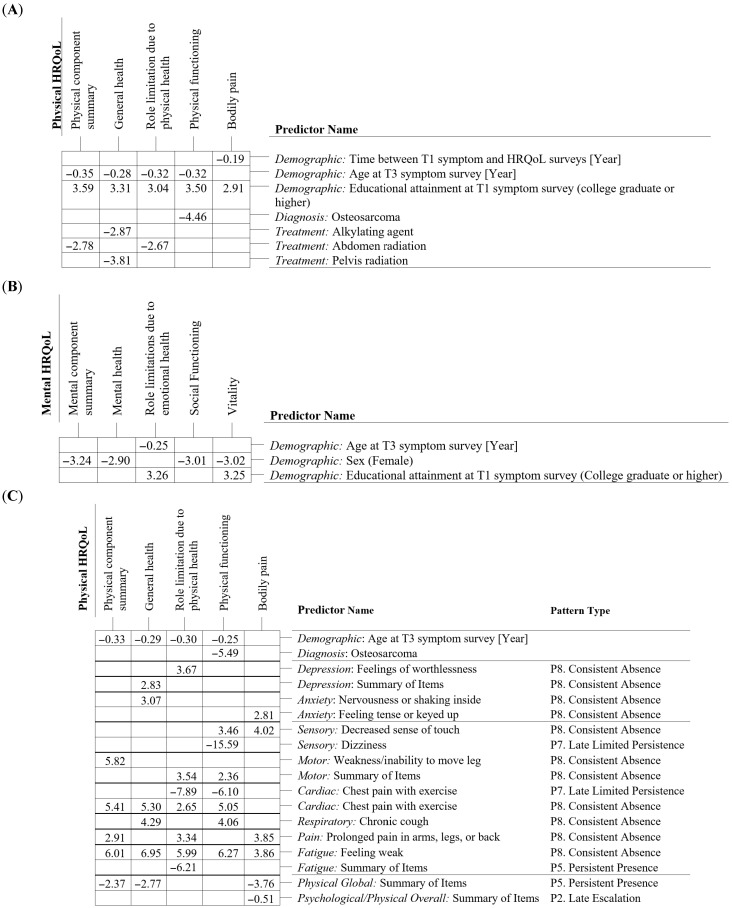

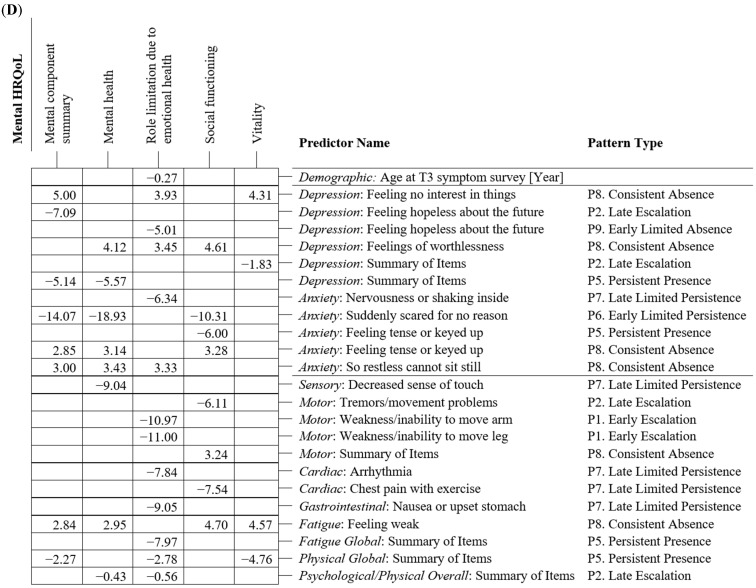
Selected predictors and estimated coefficients for HRQoL models. (**A**) Modeling physical-related HRQoL summary and domain scores, selecting predictors from the 35 non-symptom predictors. (**B**) Modeling mental-related HRQoL summary and domain scores, selecting predictors from the 35 non-symptom predictors. (**C**) Modeling physical-related HRQoL summary and domain scores, selecting predictors from the 35 non-symptom + 480 symptom change pattern predictors. (**D**) Modeling mental-related summary and domain scores, selecting predictors from the 35 non-symptom + 480 symptom change pattern predictors. Selected predictors and estimated coefficients for 576 childhood cancer survivors in (**A**) non-symptom models for physical-related HRQoL summary and domain scores; (**B**) non-symptom models for mental-related HRQoL summary and domain scores; (**C**) symptom-enhanced models for physical-related HRQoL summary and domain scores; and (**D**) symptom-enhanced models for mental-related HRQoL summary and domain scores. P1–P10 are described as follows: P1. Early escalation (i.e., a subject did not report the symptom at T1 but did at T2). P2. Late escalation (i.e., a subject did not report the symptom at T2 but did at T3). P3. Early resolution (i.e., a subject reported the symptom at T1 but not at T2). P4. Late resolution (i.e., a subject reported the symptom at T2 but not at T3). P5. Persistent presence (i.e., a subject reported the symptom at T1, T2, and T3). P6. Early limited persistence (i.e., a subject reported the symptom at T1 and T2 but not at T3). P7. Late limited persistence (i.e., a subject did not report the symptom at T1 but did at T2 and T3). P8. Consistent absence (i.e., a subject did not report the symptom at T1, T2, and T3). P9. Early limited absence (i.e., a subject did not report the symptom at T1 and T2 but did at T3). P10. Late limited absence (i.e., a subject did not report the symptom at T2 and T3 but did at T1).

Across PCS, MCS, and eight HRQoL domain scores, symptom predictors were preferentially selected over non-symptom predictors when moving from non-symptom models to symptom-enhanced models. Only age at T3 symptom assessment and a diagnosis of osteosarcoma remained selected, with coefficients similar to those in the non-symptom models (Figure 3). Two demographic predictors frequently selected in non-symptom models, i.e., having a college degree or higher (selected for seven HRQoL scores) and female sex (selected for four HRQoL scores) (Figure 3A,B), were no longer retained after incorporating symptom predictors (Figure 3C,D). Likewise, cancer treatment exposures, selected for four HRQoL scores in the non-symptom models (Figure 3A), were excluded once symptom predictors were added (Figure 3C).

Across PCS, MCS, and eight HRQoL domain scores, 77 distinct predictors were selected, including six non-symptom predictors and 71 symptom change patterns. Among these, consistent absence across all three assessments, representing 40 of the 71 selected patterns, was most frequently selected and was associated with better HRQoL. Conversely, persistent presence across all three assessments (11 change patterns) was associated with poorer HRQoL. The remaining 20 selected symptom change patterns were spread over five types: late limited persistence (eight patterns), early limited persistence (three patterns), late escalation (six patterns), early escalation (two patterns), and one early limited absence pattern. The three remaining pattern types, consistent late absence and the two resolution patterns, were never selected for any HRQoL score.

For PCS, the non-symptom model identified older age at T3 symptom assessment and prior abdominal radiation as being associated with poorer PCS, while having a college degree or higher was associated with better PCS (Table 2A). After incorporating symptom change patterns, age at T3 symptom assessment was the only non-symptom predictor that remained in the model (Table 2B). Five symptom change patterns were additionally selected, with four involving motor, cardiac, pain, and fatigue symptom domains, and one reflecting global physical symptoms. Among these, the four patterns characterized by absence were associated with better PCS, whereas the one pattern reflecting persistent presence was associated with poorer PCS. For MCS, no non-symptom predictors were retained once symptom change patterns were included. Instead, eight symptom change patterns were selected: seven related to anxiety, depression, and fatigue domains, and one reflecting global physical symptoms (Table 2D). Among these, the four absence patterns were associated with better MCS, whereas the three persistent presence patterns and the one escalation pattern were associated with poorer MCS. Across both PCS and MCS models, empirical confidence intervals obtained through bootstrap resampling were consistent with the observed effect directions. Bootstrap selection probabilities indicated lower uncertainty in variable selection for PCS than for MCS (ranging from 22% to 90% for the PCS model and from 12% to 56% for the MCS model), with confidence intervals that had zero as one of their bounds for all selected variables.

**Figure 4:**
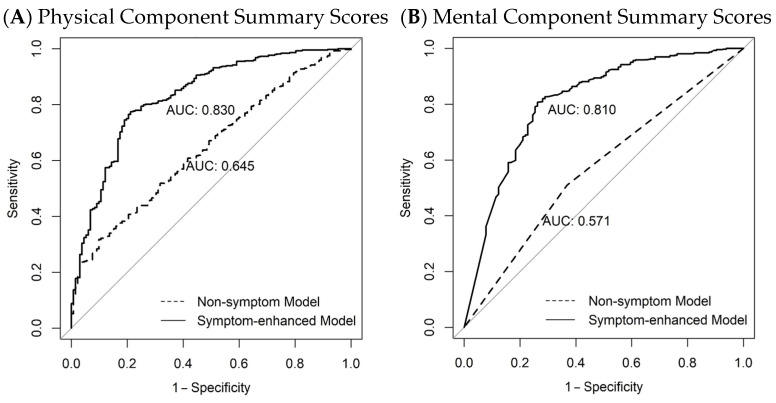
ROC curves for HRQoL models. The gray diagonal line represents the expected performance of a non-informative model (i.e., random prediction).

The transition from non-symptom to symptom-enhanced models significantly improved the prediction performance across all ten HRQoL scores, with AUC values rising to 0.74–0.85 compared with 0.56–0.66 in models lacking symptom predictors (Supplementary Table S3). For PCS, adding symptom change patterns improved performance markedly, with AUC increasing from 0.64 (cross-validation range 0.55–0.76) to 0.83 (0.70–0.95) (Figure 4 and Supplementary Table S3). For MCS, a similar improvement was observed, with the AUC increasing from 0.57 (cross-validation range 0.44–0.73) to 0.81 (0.63–0.91) (Figure 4 and Supplementary Table S3).

Receiver operating characteristic (ROC) curves for (A) physical component summary scores and (B) mental component summary scores of SF-36 for 576 childhood cancer survivors, comparing models without symptom change patterns (non-symptom model, dashed line) and with symptom change patterns (symptom-enhanced model, solid line). Predictors for the non-symptom model are selected from 35 non-symptom predictors. Predictors for the symptom-enhanced model are selected from 35 non-symptom + 480 longitudinal symptom change pattern predictors. The ROC curves and the area under the ROC curve (AUC) values are based on a cut-off of 40, representing suboptimal HRQoL.

## 4. Discussion

The key finding of this study is that longitudinal symptom change patterns substantially improved the prediction of future suboptimal HRQoL among survivors of childhood cancer, suggesting that patient-reported longitudinal symptom experiences capture aspects of survivors’ quality of life that are not fully reflected by demographic and cancer diagnosis and treatment exposures. Further, this appreciable improvement highlights the potential of low-burden, routinely collectable symptom assessments to enhance risk stratification and support clinical decision-making, including prompting timely discussion/evaluation for late effects. Accordingly, these findings underscore the importance of longitudinal symptom surveillance during follow-up care and the integration of patient-reported symptoms into survivorship models and care.

Although HRQoL is influenced by a broad constellation of factors, including chronic health conditions, lifestyle behaviors, and genetic factors, where symptoms may reflect downstream manifestations of these factors, comprehensive measurement of these factors is often not feasible in routine practice. By contrast, symptom information is relatively low-cost to collect and feasible across diverse clinical and home settings. As a result, systematic symptom assessment can be implemented widely and repeatedly, providing a pragmatic and scalable foundation for improved outcome prediction and survivorship care, which can be potentially augmented by digital health tools such as wearables, mobile applications, and remote monitoring technologies. Importantly, symptom presence or worsening commonly precedes formal disease detection or signals disease progression [30,31], often serving as the primary trigger for survivors to seek medical attention. Thus, symptom monitoring may facilitate earlier clinical attention along the survivorship care continuum.

This work contributes to the extensive literature on HRQoL among childhood cancer survivors in several novel ways. First, by focusing on multiple symptoms as primary predictors, this study relies on data that can be directly assessed in routine clinical settings rather than variables limited to research studies. Second, by utilizing a broad range of longitudinal symptom change patterns, the analysis acknowledges the dynamic nature of symptom experiences and their relationship with future HRQoL. Third, to our knowledge, this study is among the first to leverage more than two decades of longitudinal symptom data to predict HRQoL. Finally, the emphasis on patient-reported measures strengthens the relevance of the findings for patient-centered care by elevating the survivor’s voice within risk prediction.

Building on this work, future research should explore the use of longitudinal symptom predictors to model other survivorship outcomes, such as the onset or progression of CHCs, functional disability, healthcare utilization, and mortality. In parallel, the increasing integration of digital health technologies, such as mobile applications, wearable devices, and remote monitoring tools, into clinical practice is making the routine collection of continuous, real-time symptom predictors increasingly feasible. Leveraging such rich symptom predictors in future research may offer valuable insights regarding long-term health outcomes among childhood cancer survivors and support the development of more personalized survivorship care plans. In addition, while this study relied on investigator-defined symptom change patterns, automated feature generation approaches represent a promising complementary direction [32,33]. Such methods may uncover latent, complex temporal features not readily specified a priori and could further enhance predictive performance.

Several limitations should be acknowledged. First, symptom assessments were limited to predefined survey time points because of the use of secondary data. More frequent or continuous symptom monitoring could allow timely and reliable detection of symptom changes. Second, two HRQoL domains used to calculate both PCS and MCS scores involve the concept of pain, fatigue, and psychological distress, potentially creating overlap with some symptom predictors. Nonetheless, strong performance in predicting the remaining six HRQoL domains suggests that comparable performance for PCS and MCS can likely be achieved. Third, as in all long-term cohort studies, selection bias arising from non-participation and non-response is possible. Prior evaluations of the two parent cohorts found no substantial differences in demographic and cancer-related characteristics between participants and eligible non-participants, reducing concerns about major selective non-participation at cohort entry [34,35]. However, differences may still exist between survivors who were ultimately included in the present analysis and those who were not. Specifically, since inclusion in this study required completion of all three symptom assessments as well as the subsequent HRQoL assessment, differential participation across diagnostic groups is plausible. Notably, survivors of central nervous system tumors were underrepresented in this study (5.0%) compared with their representation in the parent cohorts (17.5% in CCSS and 18.0% in SJLIFE), suggesting some degree of participation bias. Dropouts among initial participants are an additional concern. Finally, chronic health conditions were not explicitly incorporated into the predictive models, despite their known associations with both symptoms and HRQoL. This reflects the study’s deliberate focus on symptom-based prediction and the practical challenges of comprehensively capturing chronic health condition data over extended follow-up periods. Given these potential limitations, caution is warranted in interpreting our results.

## 5. Conclusions

Longitudinal symptom change patterns derived from patient-reported data meaningfully improved the prediction of future HRQoL among childhood cancer survivors beyond traditional demographic and treatment-related predictors. These findings [34] highlight the potential value of routine longitudinal symptom assessment as a low-burden and scalable approach for identifying survivors at risk of poor quality of life outcomes. As survivorship populations grow and validated symptom assessment measures become increasingly available, symptom-informed models may play a critical role in delivering timely, individualized, and patient-centered survivorship care, especially with the increasing prevalence of digital monitoring tools.

## Figures and Tables

**Table 1:** Demographic, cancer diagnosis, and treatment characteristics of study participants (*n* = 576).

Predictor	Number (%) or Median (IQR)
Demographic data	
Age at T1 Symptom Survey [Year]	26.7 (23.0–30.4)
Time between T1 Symptom and HRQoL Surveys [Year]	18.9 (7.8–20.1)
Age at T3 Symptom Survey [Year]	39.5 (34.8–44.6)
Time between T3 Symptom and HRQoL Surveys [Year]	2.3 (1.5–3.8)
Age at Cancer Diagnosis [Year]	9.3 (4.5–14.1)
Sex (Female)	298 (51.7%)
Race (White)	517 (89.8%)
Educational Attainment at T1 Symptom Survey (college graduate or higher)	193 (33.5%)
Cancer Diagnosis	
Leukemia	234 (40.6%)
Hodgkin lymphoma	115 (20.0%)
Non-Hodgkin lymphoma	55 (9.5%)
Osteosarcoma	44 (7.6%)
Wilms tumor	36 (6.2%)
Central nervous system tumors	29 (5.0%)
Neuroblastoma	24 (4.2%)
Other malignancy	39 (6.8%)
Cancer Treatment	
Chemotherapy Exposure	
Plant alkaloid	448 (77.8%)
Alkylating agent	373 (64.8%)
Anthracycline	342 (59.4%)
Corticosteroid	319 (55.4%)
Methotrexate	307 (53.3%)
Intrathecal methotrexate	255 (44.3%)
Cytarabine	177 (30.7%)
High-dose methotrexate	132 (22.9%)
Intrathecal cytarabine	105 (18.2%)
Platinum	37 (6.4%)
Bleomycin	35 (6.1%)
High-dose cytarabine	20 (3.5%)
Radiation Exposure	
Brain radiation	234 (40.6%)
Chest radiation	192 (33.3%)
Neck radiation	187 (32.5%)
Abdomen radiation	161 (28.0%)
Pelvis radiation	140 (24.3%)
Surgery	
Amputation	31 (5.4%)
Other surgery	320 (55.6%)

Note: The shading is used to distinguish the major predictor categories. Abbreviations: IQR = Interquartile range.

**Table 2:** Selected models by BIEN for the SF-36’s physical and mental component summary scores without and with incorporating symptom change patterns.

Physical Component Summary					
A. Non-symptom Model (selecting predictors from the 35 non-symptom predictors)					
Predictor Name		Estimate *	95% CI *		Bootstrap Selection % †
			Lower	Upper	
(Intercept)		61.09			
*Demographic*: Age at T3 Symptom Survey [Year]		−0.35	−0.45	0.00	90%
*Demographic*: Educational Attainment at T1 Symptom Survey (college graduate or higher)		3.59	0.00	5.38	83%
*Treatment*: Abdomen radiation		−2.78	−4.6	0.00	31%
B. Symptom Model (selecting predictors from the 35 clinical predictors + 480 symptom change patterns)					
Predictor Name	Pattern Type	Estimate *	95% CI *		Bootstrap Selection % †
			Lower	Upper	
(Intercept)		46.10			
*Demographic*: Age at T3 Symptom Survey [Year]		−0.33	−0.38	0.00	90%
*Motor*: Weakness/inability to move leg	P8. Consistent Absence	5.82	0.00	7.50	39%
*Cardiac*: Chest pain with exercise	P8. Consistent Absence	5.41	0.00	6.84	86%
*Pain*: Prolonged pain in arms, legs, or back	P8. Consistent Absence	2.91	0.00	4.54	42%
*Fatigue*: Feeling weak	P8. Consistent Absence	6.01	0.00	8.03	86%
*Physical*: Summary of Items	P5. Persistent Presence	−2.37	−3.58	0.00	22%
**Mental Component Summary**					
C. Non-symptom Model (selecting predictors from the 35 non-symptom predictors)					
Predictor Name		Estimate *	95% CI *		Bootstrap Selection % †
			Lower	Upper	
(Intercept)		50.13			
*Demographic:* Sex (Female)		−3.24	−5.05	0.00	82%
D. Symptom Model (selecting predictors from the 35 clinical predictors + 480 symptom change patterns)					
Predictor Name	Pattern Type	Estimate *	95% CI *		Bootstrap Selection % †
			Lower	Upper	
(Intercept)		40.65			
*Depression*: Feeling no interest in things	P8. Consistent Absence	5.00	0.00	6.26	45%
*Depression*: Feeling hopeless about the future	P2. Late Escalation	−7.09	−8.88	0.00	23%
*Depression*: Summary of Items *	P5. Persistent Presence	−5.14	−9.71	0.00	37%
*Anxiety*: Suddenly scared for no reason	P6. Early Limited Persistence	−14.07	−22.58	0.00	56%
*Anxiety*: Feeling tense or keyed up	P8. Consistent Absence	2.85	0.00	3.96	28%
*Anxiety*: So restless cannot sit still	P8. Consistent Absence	3.00	0.00	4.80	29%
*Fatigue*: Feeling weak	P8. Consistent Absence	2.84	0.00	3.86	12%
*Physical*: Summary of Items *	P5. Persistent Presence	−2.27	−3.07	0.00	15%

Note: * Estimates are based on the original dataset. † Bootstrap selection probabilities and confidence intervals are derived from 1000 bootstrap samples. Selected pattern types are described as follows: P2. Late escalation (i.e., a subject did not report the symptom at T2 but did at T3); P5. persistent presence (i.e., a subject reported the symptom at T1, T2, and T3); P6. early limited persistence (i.e., a subject reported the symptom at T1 and T2 but not at T3); P8. consistent absence (i.e., a subject did not report the symptom at T1, T2, or T3). Predictor names consist of two parts: (1) italicized text indicating the broader category to which the predictor in each row belongs, thereby facilitating a more holistic and easier-to-interpret understanding of the results; and (2) non-italicized text stating the specific non-symptom predictor, the individual symptom, or whether the predictor represents a summary of items within the broader category. The three levels of shading indicate the Physical/Mental Component Summary outcomes, Non-symptom/Symptom candidate predictor sets, and table column information, respectively. Abbreviations: CI = Confidence interval.

## Data Availability

The dataset and analysis codes supporting the findings of this study will be made available upon acceptance of the manuscript.

## Supplementary Materials

The following supporting information can be downloaded at: https://www.mdpi.com/article/10.3390/cancers18101546/s1, Figure S1: The range and median of completion times for the three symptom surveys and the health-related quality of life survey; Figure S2: Analysis Design Overview; Figure S3: Visual representation of feature engineering process; Figure S4: Conceptual overview of the BIEN framework; Table S1: Prevalence of 48 symptom predictors among study participants at three survey time points of T1 (~1996), T2 (~2008), and T3 (~2013); Table S2: Prevalence of longitudinal symptoms change pattern among study participants over three survey time points of T1 (~1996), T2 (~2008), and T3 (~2013); Table S3: Performance evaluation for the 10 HRQoL scores modeled without incorporating symptom change patterns (non-symptom model) and with incorporating symptom change patterns (symptom-enhanced model).

## Abbreviations

AUC	Area Under the Receiver Operating Characteristic Curve
BIEN	Bayesian Information Criterion Elastic Net
CCSS	Childhood Cancer Survivor Study
CI	Confidence Interval
HRQoL	Health-Related Quality of Life
IQR	Interquartile Range
MCS	Mental Component Summary
PCS	Physical Component Summary
ROC	Receiver Operating Characteristic
SF-36	36-Item Short Form Health Survey
SJLIFE	St. Jude Lifetime Cohort Study
