# Supplementary material for: Longitudinal Patient-Reported Symptom Change Patterns and Prediction of Future Health-Related Quality of Life in Childhood Cancer Survivors: A Machine Learning Approach from the Childhood Cancer Survivor Study and the St. Jude Lifetime Cohort
Source: Cancers (Basel). 2026 May 10;18(10):1546. doi: 10.3390/cancers18101546 (PMC13204071; doi:10.3390/cancers18101546)
Supplement: Supplementary file 1 [file cancers-18-01546-s001.zip › cancers-4228308-supplementary.pdf]

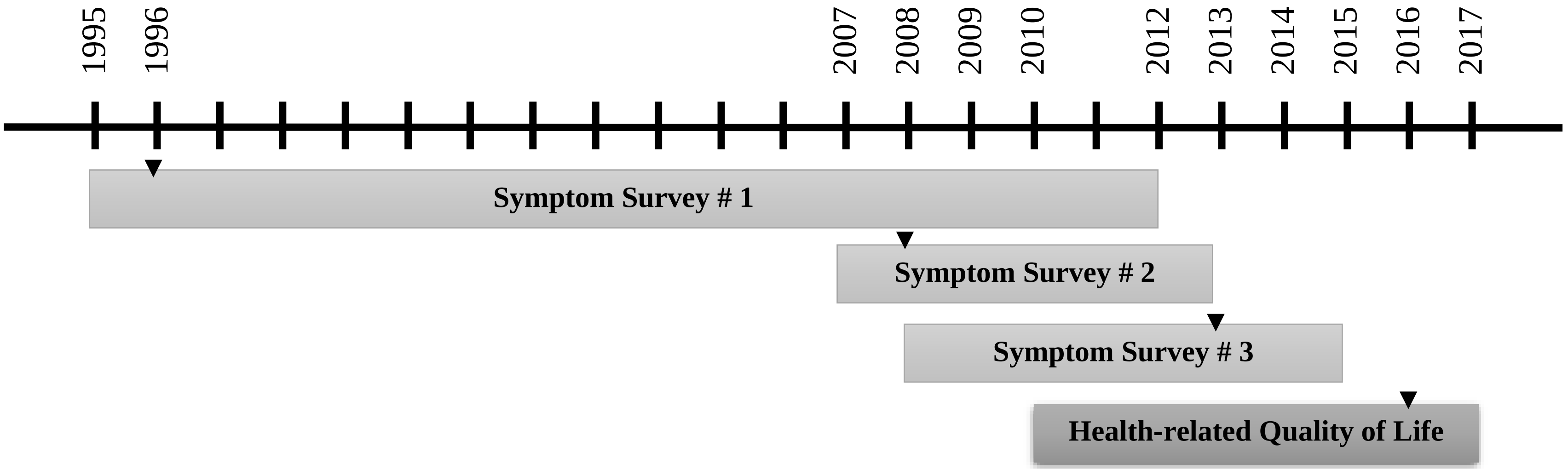


Supplementary Figure S1: The range and median of completion times for the three symptom surveys and the health-related quality of life survey.

Median values are represented by triangles. The HRQoL survey for each individual survivor was assessed subse-quent to the final symptom survey to prevent any temporal overlap between predictors and outcomes.

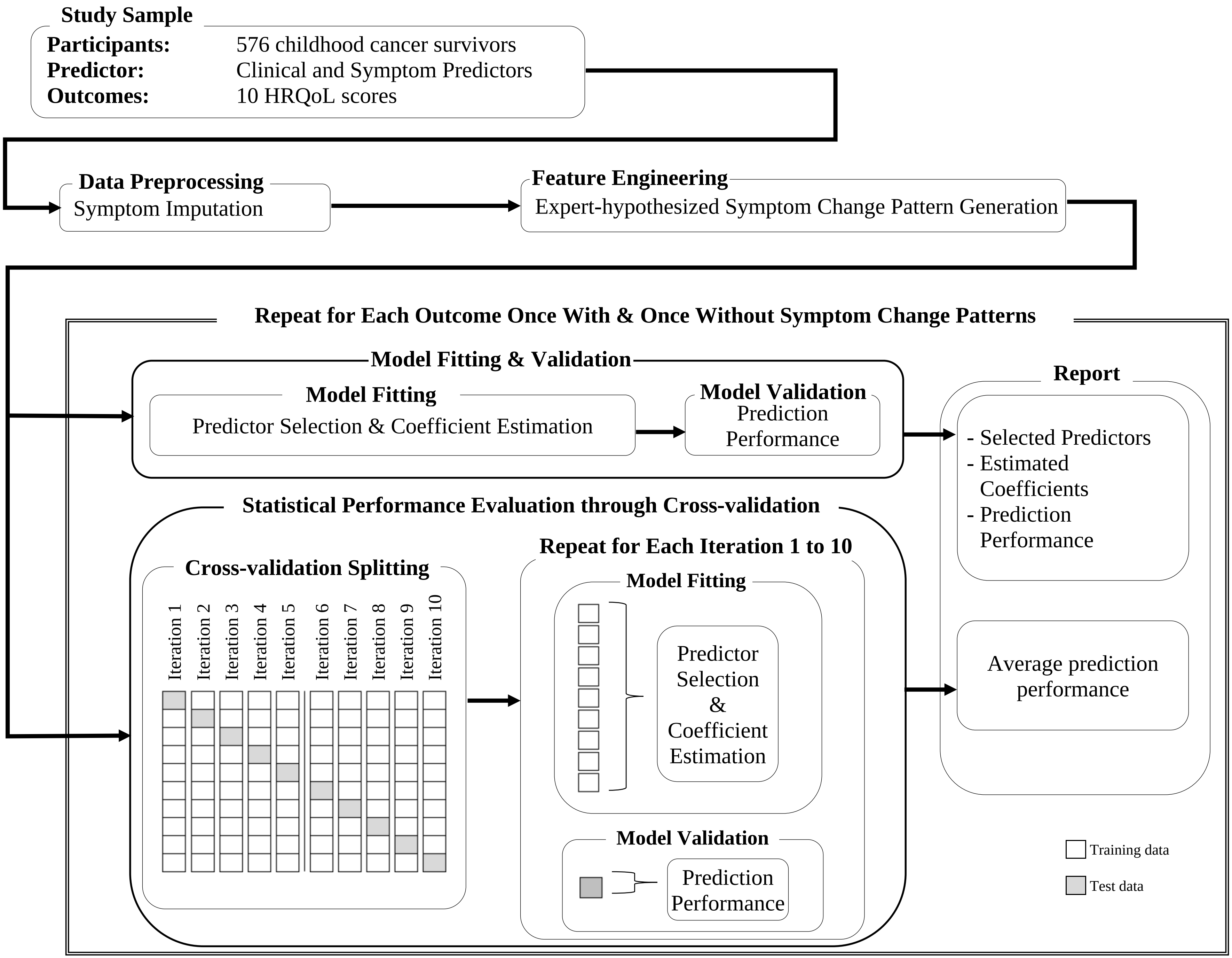


Supplementary Figure S2: Analysis Design Overview

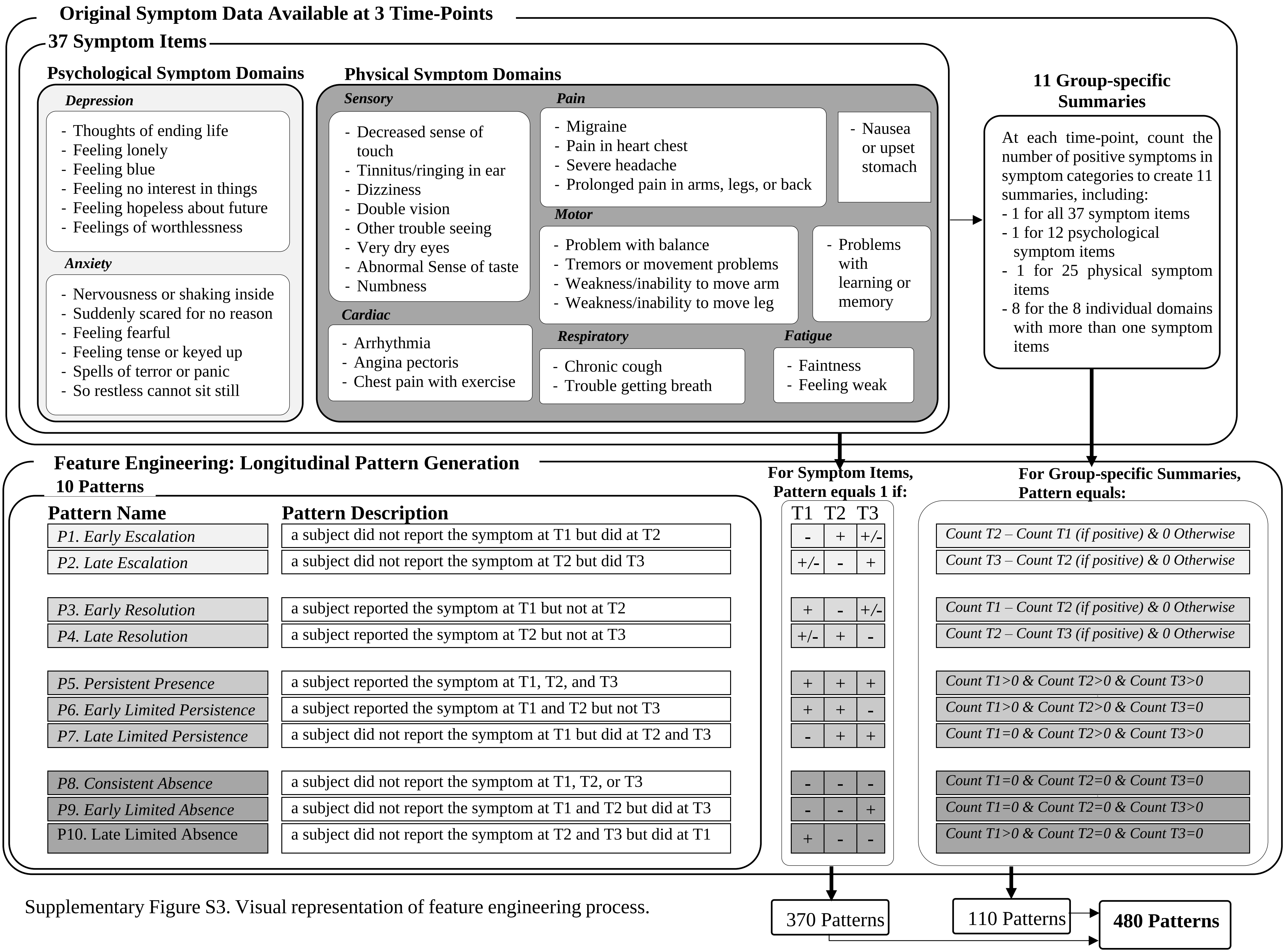


Supplementary Figure S3. Visual representation of feature engineering process.

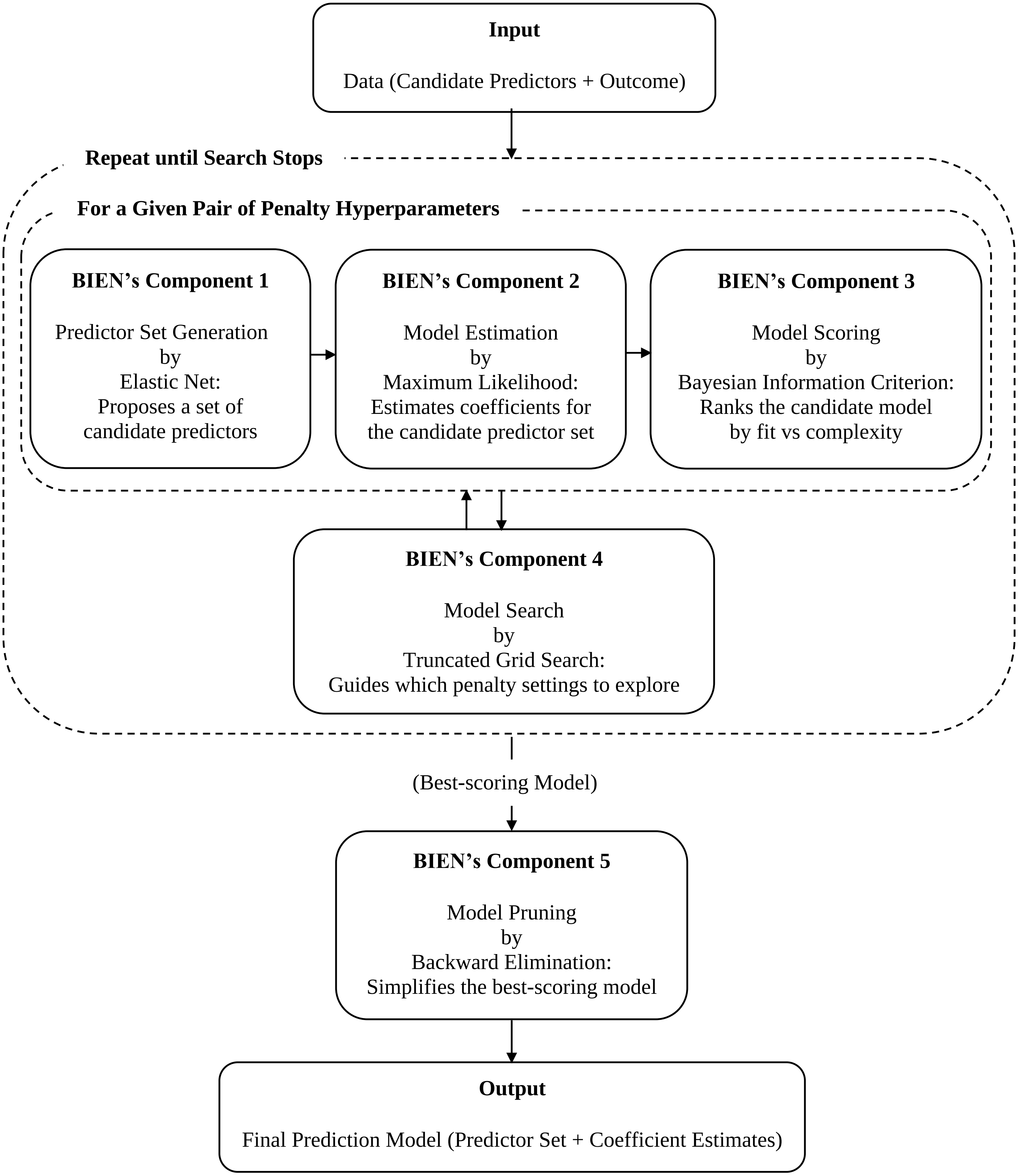


Supplementary Figure S4: Conceptual overview of the BIEN framework.

A pair of hyperparameter values determines the magnitude and the form of regularization/penalty applied to control model complexity.

Supplementary Table S1: Prevalence of 48 symptom predictors among study participants at three survey time points of T1 (~1996), T2 (~2008), and T3 (~2013)

| | Number (%) or Mean (Standard Deviation) at | | |
|---|---|---|---|
| ***Predictor (Category:* subcategory)** | **T1 (~1996)** | **T2 (~2008)** | **T3 (~2013)** |
| **Psychological Symptoms** | | | |
| 1 *Depression:* Thoughts of ending life | 9 (1.6%) | 8 (1.4%) | 5 (0.9%) |
| 2 *Depression:* Feeling lonely | 81 (14.1%) | 78 (13.5%) | 99 (17.2%) |
| 3 *Depression:* Feeling blue | 85 (14.8%) | 87 (15.1%) | 110 (19.1%) |
| 4 *Depression:* Feeling no interest in things | 55 (9.5%) | 82 (14.2%) | 88 (15.3%) |
| 5 *Depression:* Feeling hopeless about the future | 50 (8.7%) | 63 (10.9%) | 71 (12.3%) |
| 6 *Depression:* Feelings of worthlessness | 37 (6.4%) | 43 (7.5%) | 59 (10.2%) |
| 7 *Depression:* Summary of Items* | 0.6 (1.2) | 0.6 (1.3) | 0.8 (1.4) |
| 8 *Anxiety:* Nervousness or shaking inside | 54 (9.4%) | 64 (11.1%) | 86 (14.9%) |
| 9 *Anxiety:* Suddenly scared for no reason | 29 (5.0%) | 30 (5.2%) | 37 (6.4%) |
| 10 *Anxiety:* Feeling fearful | 47 (8.2%) | 50 (8.7%) | 55 (9.5%) |
| 11 *Anxiety:* Feeling tense or keyed up | 112 (19.4%) | 114 (19.8%) | 131 (22.7%) |
| 12 *Anxiety:* Spells of terror or panic | 30 (5.2%) | 35 (6.1%) | 43 (7.5%) |
| 13 *Anxiety:* So restless cannot sit still | 63 (10.9%) | 75 (13.0%) | 65 (11.3%) |
| 14 *Anxiety:* Summary of Items* | 0.6 (1.2) | 0.6 (1.3) | 0.7 (1.4) |
| **Physical Symptoms** | | | |
| 15 *Sensory:* Decreased sense of touch | 27 (4.7%) | 44 (7.6%) | 54 (9.4%) |
| 16 *Sensory:* Tinnitus/ringing in ear | 39 (6.8%) | 57 (9.9%) | 80 (13.9%) |
| 17 *Sensory:* Dizziness | 21 (3.6%) | 23 (4.0%) | 35 (6.1%) |
| 18 *Sensory:* Double vision | 13 (2.3%) | 7 (1.2%) | 12 (2.1%) |
| 19 *Sensory:* Other trouble seeing | 22 (3.8%) | 24 (4.2%) | 47 (8.2%) |
| 20 *Sensory:* Very dry eyes | 43 (7.5%) | 50 (8.7%) | 52 (9.0%) |
| 21 *Sensory:* Abnormal Sense of taste | 11 (1.9%) | 7 (1.2%) | 8 (1.4%) |
| 22 *Sensory:* Numbness | 48 (8.3%) | 78 (13.5%) | 108 (18.8%) |
| 23 *Sensory:* Summary of Items* | 0.4 (0.8) | 0.5 (0.9) | 0.7 (1.0) |
| *24 Motor:* Problem with balance | 35 (6.1%) | 51 (8.9%) | 75 (13.0%) |
| *25 Motor:* Tremors/movement problems | 19 (3.3%) | 13 (2.3%) | 22 (3.8%) |
| *26 Motor:* Weakness/inability to move arm | 27 (4.7%) | 24 (4.2%) | 33 (5.7%) |
| *27 Motor:* Weakness/inability to move leg | 26 (4.5%) | 18 (3.1%) | 30 (5.2%) |
| *28 Motor:* Summary of Items* | 0.2 (0.6) | 0.2 (0.5) | 0.3 (0.7) |
| *29 Cardiac:* Arrhythmia | 28 (4.9%) | 40 (6.9%) | 55 (9.5%) |
| *30 Cardiac:* Angina pectoris | 2 (0.3%) | 3 (0.5%) | 7 (1.2%) |
| *31 Cardiac:* Chest pain with exercise | 72 (12.5%) | 65 (11.3%) | 96 (16.7%) |
| *32 Cardiac:* Summary of Items* | 0.2 (0.4) | 0.2 (0.5) | 0.3 (0.6) |
| *33 Respiratory:* Chronic cough | 34 (5.9%) | 31 (5.4%) | 39 (6.8%) |
| *34 Respiratory:* Trouble getting breath | 33 (5.7%) | 31 (5.4%) | 57 (9.9%) |
| *35 Respiratory:* Summary of Items* | 0.1 (0.4) | 0.1 (0.4) | 0.2 (0.4) |
| *36 Memory:* Problems with learning or memory | 76 (13.2%) | 129 (22.4%) | 173 (30.0%) |
| *37 Pain:* Migraine | 97 (16.8%) | 91 (15.8%) | 97 (16.8%) |
| *38 Pain:* Pain in heart chest | 20 (3.5%) | 36 (6.2%) | 44 (7.6%) |
| *39 Pain:* Severe headache | 110 (19.1%) | 83 (14.4%) | 65 (11.3%) |
| *40 Pain:* Prolonged pain in arms, legs, or back | 76 (13.2%) | 97 (16.8%) | 117 (20.3%) |
| *41 Pain:* Summary of Items* | 0.5 (0.8) | 0.5 (0.9) | 0. 6 (0.9) |
| *42 Gastrointestinal:* Nausea or upset stomach | 74 (12.8%) | 68 (11.8%) | 75 (13.0%) |
| *43 Fatigue:* Faintness | 19 (3.3%) | 27 (4.7%) | 49 (8.5%) |
| *44 Fatigue:* Feeling weak | 50 (8.7%) | 72 (12.5%) | 92 (16.0%) |
| *45 Fatigue:* Summary of Items* | 0.1 (0.4) | 0.2 (0.4) | 0.2 (0.5) |
| **Psychological and/or Physical Summaries** | | | |
| *46 Psychological Global:* Summary of Items* | 1.13 (2.22) | 1.27 (2.38) | 1.47 (2.48) |
| *47 Physical Global:* Summary of Items* | 1.77 (2.53) | 2.03 (2.70) | 2.64 (3.23) |
| *48 Psychological/Physical Overall:* Summary of Items* | 2.9 (4.0) | 3.3 (4.3) | 4.1 (5.0) |

**Note**: * Continuous measures counting positive *symptom items* in the corresponding domain

Supplementary Table S2: Prevalence of longitudinal symptoms change pattern among study participants over three survey time points of T1 (~1996), T2 (~2008), and T3 (~2013)

| | Number (%) or Mean (Standard Deviation) for Patterns† of | | | | | | | | | |
|---|---|---|---|---|---|---|---|---|---|---|
| ***Predictor (Category:* subcategory)*** | **P1: -,+,+/-** | **P2: +/-,-,+** | **P3: +,-,+/-** | **P4: +/-,+,-** | **P5: +,+,+** | **P6: +,+,-** | **P7: -,+,+** | **P8: -,-,-** | **P9**: -,-,+ | **P10**: +,-,- |
| **Psychological Symptoms** | | | | | | | | | | |
| 1 *Depression:* Thoughts of ending life | 5 (0.9%) | 5 (0.9%) | 6 (1.0%) | 8 (1.4%) | 0 (0.0%) | 3 (0.5%) | 0 (0.0%) | 557 (96.7%) | 5 (0.9%) | 6 (1.0%) |
| 2 *Depression:* Feeling lonely | 51 (8.9%) | 54 (9.4%) | 54 (9.4%) | 33 (5.7%) | 18 (3.1%) | 9 (1.6%) | 27 (4.7%) | 403 (70.0%) | 41 (7.1%) | 41 (7.1%) |
| 3 *Depression:* Feeling blue | 57 (9.9%) | 68 (11.8%) | 55 (9.5%) | 45 (7.8%) | 18 (3.1%) | 12 (2.1%) | 24 (4.2%) | 381 (66.1%) | 53 (9.2%) | 40 (6.9%) |
| 4 *Depression:* Feeling no interest in things | 57 (9.9%) | 47 (8.2%) | 30 (5.2%) | 41 (7.1%) | 15 (2.6%) | 10 (1.7%) | 26 (4.5%) | 428 (74.3%) | 36 (6.2%) | 19 (3.3%) |
| 5 *Depression:* Feeling hopeless about the future | 45 (7.8%) | 43 (7.5%) | 32 (5.6%) | 35 (6.1%) | 11 (1.9%) | 7 (1.2%) | 17 (3.0%) | 443 (76.9%) | 38 (6.6%) | 27 (4.7%) |
| 6 *Depression:* Feelings of worthlessness | 32 (5.6%) | 38 (6.6%) | 26 (4.5%) | 22 (3.8%) | 9 (1.6%) | 2 (0.3%) | 12 (2.1%) | 475 (82.5%) | 32 (5.6%) | 20 (3.5%) |
| 7 *Depression:* Summary of Items* | 0.40 (1.03) | 0.41 (1.01) | 0.32 (0.93) | 0.28 (0.82) | 42 (7.3%) | 18 (3.1%) | 45 (7.8%) | 316 (54.9%) | 57 (9.9%) | 43 (7.5%) |
| 8 *Anxiety:* Nervousness or shaking inside | 41 (7.1%) | 53 (9.2%) | 31 (5.4%) | 31 (5.4%) | 14 (2.4%) | 9 (1.6%) | 19 (3.3%) | 433 (75.2%) | 48 (8.3%) | 26 (4.5%) |
| 9 *Anxiety:* Suddenly scared for no reason | 17 (3.0%) | 25 (4.3%) | 16 (2.8%) | 18 (3.1%) | 6 (1.0%) | 7 (1.2%) | 6 (1.0%) | 508 (88.2%) | 22 (3.8%) | 13 (2.3%) |
| 10 *Anxiety:* Feeling fearful | 36 (6.2%) | 29 (5.0%) | 33 (5.7%) | 24 (4.2%) | 12 (2.1%) | 2 (0.3%) | 14 (2.4%) | 467 (81.1%) | 26 (4.5%) | 30 (5.2%) |
| 11 *Anxiety:* Feeling tense or keyed up | 66 (11.5%) | 73 (12.7%) | 64 (11.1%) | 56 (9.7%) | 32 (5.6%) | 16 (2.8%) | 26 (4.5%) | 342 (59.4%) | 56 (9.7%) | 47 (8.2%) |
| 12 *Anxiety:* Spells of terror or panic | 24 (4.2%) | 26 (4.5%) | 19 (3.3%) | 18 (3.1%) | 9 (1.6%) | 2 (0.3%) | 8 (1.4%) | 498 (86.5%) | 24 (4.2%) | 17 (3.0%) |
| 13 *Anxiety:* So restless cannot sit still | 49 (8.5%) | 37 (6.4%) | 37 (6.4%) | 47 (8.2%) | 14 (2.4%) | 12 (2.1%) | 14 (2.4%) | 433 (75.2%) | 31 (5.4%) | 31 (5.4%) |
| 14 *Anxiety:* Summary of Items* | 0.38 (0.95) | 0.39 (0.92) | 0.32 (0.79) | 0.30 (0.80) | 56 (9.7%) | 28 (4.9%) | 40 (6.9%) | 273 (47.4%) | 63 (10.9%) | 57 (9.9%) |
| **Physical Symptoms** | | | | | | | | | | |
| 15 *Sensory:* Decreased sense of touch | 33 (5.7%) | 32 (5.6%) | 16 (2.8%) | 22 (3.8%) | 6 (1.0%) | 5 (0.9%) | 16 (2.8%) | 487 (84.5%) | 29 (5.0%) | 13 (2.3%) |
| 16 *Sensory:* Tinnitus/ringing in ear | 36 (6.2%) | 37 (6.4%) | 18 (3.1%) | 14 (2.4%) | 17 (3.0%) | 4 (0.7%) | 26 (4.5%) | 468 (81.2%) | 33 (5.7%) | 14 (2.4%) |
| 17 *Sensory:* Dizziness | 14 (2.4%) | 26 (4.5%) | 12 (2.1%) | 14 (2.4%) | 5 (0.9%) | 4 (0.7%) | 4 (0.7%) | 517 (89.8%) | 24 (4.2%) | 10 (1.7%) |
| 18 *Sensory:* Double vision | 3 (0.5%) | 9 (1.6%) | 9 (1.6%) | 4 (0.7%) | 3 (0.5%) | 1 (0.2%) | 0 (0.0%) | 553 (96.0%) | 7 (1.2%) | 7 (1.2%) |
| 19 *Sensory:* Other trouble seeing | 15 (2.6%) | 35 (6.1%) | 13 (2.3%) | 12 (2.1%) | 5 (0.9%) | 4 (0.7%) | 7 (1.2%) | 507 (88.0%) | 32 (5.6%) | 10 (1.7%) |
| 20 *Sensory:* Very dry eyes | 34 (5.9%) | 30 (5.2%) | 27 (4.7%) | 28 (4.9%) | 8 (1.4%) | 8 (1.4%) | 14 (2.4%) | 477 (82.8%) | 22 (3.8%) | 19 (3.3%) |
| 21 *Sensory:* Abnormal Sense of taste | 4 (0.7%) | 6 (1.0%) | 8 (1.4%) | 5 (0.9%) | 0 (0.0%) | 3 (0.5%) | 2 (0.3%) | 555 (96.4%) | 6 (1.0%) | 8 (1.4%) |
| 22 *Sensory:* Numbness | 59 (10.2%) | 69 (12.0%) | 29 (5.0%) | 39 (6.8%) | 16 (2.8%) | 3 (0.5%) | 23 (4.0%) | 408 (70.8%) | 61 (10.6%) | 21 (3.6%) |
| 23 *Sensory:* Summary of Items* | 0.29 (0.64) | 0.38 (0.73) | 0.18 (0.47) | 0.20 (0.50) | 67 (11.6%) | 24 (4.2%) | 57 (9.9%) | 240 (41.7%) | 86 (14.9%) | 31 (5.4%) |
| 24 *Motor:* Problem with balance | 32 (5.6%) | 41 (7.1%) | 16 (2.8%) | 17 (3.0%) | 15 (2.6%) | 4 (0.7%) | 19 (3.3%) | 470 (81.6%) | 39 (6.8%) | 14 (2.4%) |
| 25 *Motor:* Tremors/movement problems | 11 (1.9%) | 19 (3.3%) | 17 (3.0%) | 10 (1.7%) | 1 (0.2%) | 1 (0.2%) | 2 (0.3%) | 529 (91.8%) | 17 (3.0%) | 15 (2.6%) |
| 26 *Motor:* Weakness/inability to move arm | 10 (1.7%) | 21 (3.6%) | 13 (2.3%) | 12 (2.1%) | 8 (1.4%) | 6 (1.0%) | 4 (0.7%) | 522 (90.6%) | 17 (3.0%) | 9 (1.6%) |
| 27 *Motor:* Weakness/inability to move leg | 10 (1.7%) | 23 (4.0%) | 18 (3.1%) | 11 (1.9%) | 5 (0.9%) | 3 (0.5%) | 2 (0.3%) | 522 (90.6%) | 18 (3.1%) | 13 (2.3%) |
| 28 *Motor:* Summary of Items* | 0.10 (0.36) | 0.17 (0.56) | 0.10 (0.40) | 0.07 (0.32) | 24 (4.2%) | 10 (1.7%) | 25 (4.3%) | 422 (73.3%) | 46 (8.0%) | 24 (4.2%) |
| 29 *Cardiac:* Arrhythmia | 25 (4.3%) | 26 (4.5%) | 13 (2.3%) | 11 (1.9%) | 12 (2.1%) | 3 (0.5%) | 17 (3.0%) | 500 (86.8%) | 23 (4.0%) | 10 (1.7%) |
| 30 *Cardiac:* Angina pectoris | 3 (0.5%) | 6 (1.0%) | 2 (0.3%) | 2 (0.3%) | 0 (0.0%) | 0 (0.0%) | 1 (0.2%) | 565 (98.1%) | 6 (1.0%) | 2 (0.3%) |
| 31 *Cardiac:* Chest pain with exercise | 38 (6.6%) | 53 (9.2%) | 45 (7.8%) | 22 (3.8%) | 23 (4.0%) | 4 (0.7%) | 20 (3.5%) | 430 (74.7%) | 36 (6.2%) | 28 (4.9%) |
| 32 *Cardiac:* Summary of Items* | 0.11 (0.35) | 0.15 (0.40) | 0.10 (0.31) | 0.06 (0.25) | 33 (5.7%) | 6 (1.0%) | 27 (4.7%) | 391 (67.9%) | 46 (8.0%) | 34 (5.9%) |
| 33 *Respiratory:* Chronic cough | 17 (3.0%) | 22 (3.8%) | 20 (3.5%) | 14 (2.4%) | 6 (1.0%) | 8 (1.4%) | 11 (1.9%) | 506 (87.8%) | 19 (3.3%) | 17 (3.0%) |
| 34 *Respiratory:* Trouble getting breath | 24 (4.2%) | 38 (6.6%) | 26 (4.5%) | 12 (2.1%) | 5 (0.9%) | 2 (0.3%) | 14 (2.4%) | 485 (84.2%) | 34 (5.9%) | 22 (3.8%) |
| 35 *Respiratory:* Summary of Items* | 0.07 (0.29) | 0.10 (0.35) | 0.07 (0.28) | 0.05 (0.23) | 15 (2.6%) | 7 (1.2%) | 17 (3.0%) | 452 (78.5%) | 38 (6.6%) | 26 (4.5%) |
| 36 *Memory:* Problems with learning or memory | 83 (14.4%) | 77 (13.4%) | 30 (5.2%) | 33 (5.7%) | 39 (6.8%) | 7 (1.2%) | 57 (9.9%) | 346 (60.1%) | 71 (12.3%) | 24 (4.2%) |
| 37 *Pain:* Migraine | 39 (6.8%) | 35 (6.1%) | 45 (7.8%) | 29 (5.0%) | 39 (6.8%) | 13 (2.3%) | 23 (4.0%) | 415 (72.0%) | 25 (4.3%) | 35 (6.1%) |
| 38 *Pain:* Pain in heart chest | 30 (5.2%) | 33 (5.7%) | 14 (2.4%) | 25 (4.3%) | 3 (0.5%) | 3 (0.5%) | 8 (1.4%) | 498 (86.5%) | 28 (4.9%) | 9 (1.6%) |
| 39 *Pain:* Severe headache | 40 (6.9%) | 29 (5.0%) | 67 (11.6%) | 47 (8.2%) | 21 (3.6%) | 22 (3.8%) | 15 (2.6%) | 412 (71.5%) | 14 (2.4%) | 52 (9.0%) |
| 40 *Pain:* Prolonged pain in arms, legs, or back | 54 (9.4%) | 59 (10.2%) | 33 (5.7%) | 39 (6.8%) | 30 (5.2%) | 13 (2.3%) | 28 (4.9%) | 401 (69.6%) | 45 (7.8%) | 19 (3.3%) |

| Predictor (Category: subcategory)* | Number (%) or Mean (Standard Deviation) for Patterns† of | | | | | | | | | |
|---|---|---|---|---|---|---|---|---|---|---|
| | **P1: -,+,+/-** | **P2: +/-,-,+** | **P3: +,-,+/-** | **P4: +/-,+,-** | **P5: +,+,+** | **P6: +,+,-** | **P7: -,+,+** | **P8: -,-,-** | **P9: -,-,+** | **P10: +,-,-** |
| *41 Pain:* Summary of Items* | 0.24 (0.59) | 0.23 (0.50) | 0.23 (0.51) | 0.20 (0.52) | 96 (16.7%) | 34 (5.9%) | 42 (7.3%) | 255 (44.3%) | 47 (8.2%) | 42 (7.3%) |
| *42 Gastrointestinal:* Nausea or upset stomach | 47 (8.2%) | 51 (8.9%) | 53 (9.2%) | 44 (7.6%) | 13 (2.3%) | 8 (1.4%) | 11 (1.9%) | 421 (73.1%) | 34 (5.9%) | 36 (6.2%) |
| *43 Fatigue:* Faintness | 22 (3.8%) | 37 (6.4%) | 14 (2.4%) | 15 (2.6%) | 1 (0.2%) | 4 (0.7%) | 11 (1.9%) | 500 (86.8%) | 35 (6.1%) | 12 (2.1%) |
| *44 Fatigue:* Feeling weak | 47 (8.2%) | 50 (8.7%) | 25 (4.3%) | 30 (5.2%) | 20 (3.5%) | 5 (0.9%) | 22 (3.8%) | 436 (75.7%) | 43 (7.5%) | 18 (3.1%) |
| *45 Fatigue:* Summary of Items* | 0.11 (0.35) | 0.14 (0.41) | 0.06 (0.25) | 0.07 (0.28) | 23 (4.0%) | 7 (1.2%) | 30 (5.2%) | 409 (71.0%) | 51 (8.9%) | 19 (3.3%) |
| **Psychological and/or Physical Summaries** | | | | | | | | | | |
| *46 Psychological Global:* Summary of Items* | 0.73 (1.74) | 0.74 (1.66) | 0.60 (1.49) | 0.53 (1.40) | 88 (15.3%) | 30 (5.2%) | 59 (10.2%) | 233 (40.5%) | 56 (9.7%) | 48 (8.3%) |
| *47 Physical Global:* Summary of Items* | 0.88 (1.68) | 1.12 (1.90) | 0.63 (1.25) | 0.51 (1.16) | 238 (41.3%) | 27 (4.7%) | 72 (12.5%) | 77 (13.4%) | 50 (8.7%) | 38 (6.6%) |
| *48 Psychological/Physical Overall:* Summary of Items* | 1.46 (2.75) | 1.67 (2.91) | 1.07 (2.24) | 0.85 (1.94) | 279 (48.4%) | 36 (6.2%) | 62 (10.8%) | 51 (8.9%) | 46 (8.0%) | 33 (5.7%) |

**Note**: * For summary measures, P1-P4 (i.e., escalation/resolution) patterns are continuous determined based on the counts of present *symptom items*, while P5-P10 (i.e., consistency patterns) are binary and determined based on the presence of any symptoms (i.e., count > 0), † Patterns, denoted as P1-P10, are characterized by three signs indicators representing symptom status at T1, T2, and T3, where + indicates symptom presence, - indicates symptom absence, and +/- indicates that symptom may be present or absent. P1-P10 are described as follows: P1. Early Escalation (i.e., a subject did not report the symptom at T1 but did at T2); P2. Late Escalation (i.e., a subject did not report the symptom at T2 but did at T3); P3. Early Resolution (i.e., a subject reported the symptom at T1 but not at T2); P4. Late Resolution (i.e., a subject reported the symptom at T2 but not at T3); P5. Persistent Presence (i.e., a subject reported the symptom at T1, T2, and T3); P6. Early Limited Persistence (i.e., a subject reported the symptom at T1 and T2 but not at T3); P7. Late Limited Persistence (i.e., a subject did not report the symptom at T1 but did at T2 and T3); P8. Consistent Absence (i.e., a subject did not report the symptom at T1, T2, or T3); P9. Early Limited Absence (i.e., a subject did not report the symptom at T1 and T2 but did at T3); P10. Late Limited Absence (i.e., a subject did not report the symptom at T2 and T3 but did at T1).

Supplementary Table S3: Performance evaluation for the 10 HRQoL scores modelled without incorporating symptom change patterns (non-symptom model) and with incorporating symptom change patterns (symptom-enhanced model)

| | **Non-symptom model** | | **Symptom-enhanced Model** | | **P-value§** |
|---|---|---|---|---|---|
| | **Model AUC*†** | **Cross-validated AUC*‡** | **Model AUC*†** | **Cross-validated AUC*‡** | |
| **Physical Component Score** | 0.645 | 0.626 (0.547, 0.760) | 0.830 | 0.797 (0.696, 0.950) | <0.001 |
| General health perception | 0.632 | 0.583 (0.482, 0.672) | 0.826 | 0.792 (0.709, 0.856) | <0.001 |
| Role limitation due to physical health | 0.654 | 0.608 (0.509, 0.790) | 0.807 | 0.781 (0.672, 0.906) | <0.001 |
| Physical functioning | 0.661 | 0.650 (0.522, 0.815) | 0.836 | 0.801 (0.671, 0.847) | <0.001 |
| Bodily pain | 0.585 | 0.566 (0.441, 0.706) | 0.794 | 0.767 (0.644, 0.944) | <0.001 |
| **Mental Component Score** | 0.571 | 0.559 (0.445, 0.730) | 0.810 | 0.743 (0.626, 0.909) | <0.001 |
| Mental health | 0.556 | 0.558 (0.395, 0.710) | 0.809 | 0.739 (0.582, 0.909) | <0.001 |
| Role limitation due to emotional health | 0.642 | 0.612 (0.435, 0.800) | 0.854 | 0.800 (0.715, 0.859) | <0.001 |
| Social functioning | 0.577 | 0.559 (0.442, 0.655) | 0.785 | 0.752 (0.594, 0.885) | <0.001 |
| Vitality | 0.577 | 0.541 (0.441, 0.675) | 0.745 | 0.696 (0.448, 0.854) | <0.001 |

**Note**: * Area Under the Receiver Operating Characteristic Curve (AUC), calculated using a cutoff of 40 to indicate clinically meaningful impairment; † Model AUC represents the AUC value obtained from fitting and validating the model in the entire study sample; ‡ Cross-validated AUC mean (range across 10 cross-validation iterations) is the average AUC value (along with the range) obtained from the 10 cross-validation iterations; § P-value is reported based on DeLong's test comparing the two Receiver Operating Characteristic curves obtained from the entire study sample, one without the symptom predictors (non-symptom model) and one with the symptom predictors (symptom-enhanced model).
